# Biomechanical Effects of a Cross Connector in Sacral Fractures – A Finite Element Analysis

**DOI:** 10.3389/fbioe.2021.669321

**Published:** 2021-05-26

**Authors:** Meike Gierig, Fangrui Liu, Lukas Weiser, Wolfgang Lehmann, Peter Wriggers, Michele Marino, Dominik Saul

**Affiliations:** ^1^Institute of Continuum Mechanics, Leibniz University Hannover, Hanover, Germany; ^2^Department of Trauma, Orthopedics and Reconstructive Surgery, Georg-August-University of Göttingen, Göttingen, Germany; ^3^Department of Civil Engineering and Computer Science, University of Rome Tor Vergata, Rome, Italy; ^4^Kogod Center on Aging and Division of Endocrinology, Mayo Clinic, Rochester, MN, United States

**Keywords:** spinopelvic fracture, sacrum fracture, spinopelvic stabilization, finite element analysis, cross connector

## Abstract

**Background:** Spinopelvic fractures and approaches of operative stabilization have been a source of controversial discussion. Biomechanical data support the benefit of a spinopelvic stabilization and minimally invasive procedures help to reduce the dissatisfying complication rate. The role of a cross connector within spinopelvic devices remains inconclusive. We aimed to analyze the effect of a cross connector in a finite element model (FE model).

**Study Design:** A FE model of the L1-L5 spine segment with pelvis and a spinopelvic stabilization was reconstructed from patient-specific CT images. The biomechanical relevance of a cross connector in a Denis zone I (AO: 61-B2) sacrum fracture was assessed in the FE model by applying bending and twisting forces with and without a cross connector. Biomechanical outcomes from the numerical model were investigated also considering uncertainties in material properties and levels of osseointegration.

**Results:** The designed FE model showed comparable values in range-of-motion (ROM) and stresses with reference to the literature. The superiority of the spinopelvic stabilization (L5/Os ilium) ± cross connector compared to a non-operative procedure was confirmed in all analyzed loading conditions by reduced ROM and principal stresses in the disk L5/S1, vertebral body L5 and the fracture area. By considering the combination of all loading cases, the presence of a cross connector reduced the maximum stresses in the fracture area of around 10%. This difference has been statistically validated (*p* < 0.0001).

**Conclusion:** The implementation of a spinopelvic stabilization (L5/Os ilium) in sacrum fractures sustained the fracture and led to enhanced biomechanical properties compared to a non-reductive procedure. While the additional cross connector did not alter the resulting ROM in L4/L5 or L5/sacrum, the reduction of the maximum stresses in the fracture area was significant.

## Introduction

Representing the anatomical connection between the spine and pelvis, the sacrum acts as a biomechanical keystone. Anteriorly directed axial forces from the spinal column, the body weight (downward directed) and resistance to the ground (upward directed) act on the sacrum and its ligamentous fixation (Rizkalla et al., 2019). But the constant force transmission to the lower extremities makes the sacrum a highly stressed bone, with clearly defined weak points along the sacral foramina, notably prone to fragility fractures (Pascal-Moussellard et al., 2016; Borgström et al., 2020). Sacrum fractures lead to a severe pattern of injury which is highly unstable and associated with sacral nerve root injury, severe bleeding and soft tissue damage (Williams and Quinnan, 2016). The treatment of these injuries always has been a root of controversial discussion. Both the decision on whether to operate and on the way of stabilization have been diversely debated (Williams and Quinnan, 2016; Guerado et al., 2018). Loss of reduction in 26% and a malunion rate of 44% clarify that with a single iliosacral screw alone, the vertically unstable pelvis is not sufficiently treated (Keating et al., 1999).

Focusing on complex sacrum fractures, two entities need to be discerned, the osteoporotic fragility fracture and the high-energy fracture in the young. Since the fragility fractures of the pelvis have been extensively described by Rommens et al. (2015), they are getting more into the focus of trauma surgeons in developed countries due to the demographic transformation (Borgström et al., 2020). The osteoporotic sacral insufficiency fracture is reported with an incidence of 1–5% (Tsiridis et al., 2006). In this special multimorbid collective, surgical intervention must be narrowed down to the outright essential.

Apart from that, the complex traumatic sacrum fracture beside the rare entity of spinopelvic discontinuation in severe trauma patients depict utterly different fracture patterns. The traumatic central sacrum fractures are a condition that can be stated generally rare with an incidence of 2 per 100, 000 (Beckmann and Chinapuvvula, 2017), while within pelvic trauma patients, the unstable sacral fracture has an incidence of 17–30% (Jazini et al., 2017a). Regarding the operative treatment, complex fractures cannot adequately be reduced by only iliosacral screws but need vertical support and the neutralization of shearing forces (Guerado et al., 2018). In the mostly young patient with proper bone, the early mobilization and load/weight bearing are main factors to aim at in the operative therapy, while in the osteoporotic patient the immediate mobilization is crucial for the long-term outcome (Williams and Quinnan, 2016; Pulley et al., 2018).

The surgical technique that is mostly favored in complex spinopelvic injuries is the spinopelvic fixation from L4/5 to the ilium or the spinopelvic fixation from L4/5 to the ilium with a cross connector (CC). In terms of H-fractures of the sacrum, the CC is thought to stabilize the fracture components and prevent further discontinuation from the spine to the pelvis. After a posterior or anteroposterior stabilization, a rapid fusion of the bone and short postoperative immobilization of the patient are intended in order to avoid immobilization-induced complications (Wagner et al., 2015). This leads to a delicate balance between the stability of the construct and potentially occurring material fatigue (Melkerson, 2003).

While the biomechanically favorable impact of the lumbopelvic stabilization has been proven, the addition of a CC has not been biomechanically assessed to date. Nonetheless, there is reliable data that bilateral stabilization is necessary to immobilize the sacroiliac joint (Jazini et al., 2017a; Lindsey et al., 2018). In theory, after proximal L5 fixation, shearing and rotating forces could be addressed by cross connectors (Guerado et al., 2018). Thus, in lumbopelvic instrumentation, the addition of a cross-connecting device was mentioned by Bellabara et al. to “further stabilize … [the] hemipelvis” (Bellabarba et al., 2006). Similarly, a new minimally invasive approach that was introduced by the Hannover group used percutaneous L3 and L4 as well as iliac screws with long rods, connected with a 5.5 mm crossing rod in order to reach a “high construct rigidity” in lumbosacral fractures (Decker et al., 2019). The biomechanical proof of that rigidity remains to be elucidated.

If the surgeon decides to add a CC to his spinopelvic construct, he has to consider two facts. First, that there is no biomechanical approval of this concept. Second, the raised level of infection rate after the utilization of a CC with a reported rate of postoperative infection and healing disturbances around 16-38% due to the larger incision (Bellabarba et al., 2006; Schildhauer et al., 2006; König et al., 2012). There has been a long-lasting debate on the reasons of infection after spinopelvic- and cross-connector-application in spinal surgery, even after minimally invasive approaches have been widely established (Bellabarba et al., 2006; Williams and Quinnan, 2016; Barcellos et al., 2017; Jazini et al., 2017b; Decker et al., 2019). This is due to the preparative extent for an insertion of a cross connector, as it is challenging to insert it in a minimally invasive way. In cases of spinal decompression, the extensive approach is done either way, but otherwise the lumbopelvic construct can also be introduced in a minimally invasive approach without cross connectors. The price of additional stabilization comes with the danger of revision surgery, which especially in the elderly population can be a life-threatening term.

To determine the value of a CC in the clinical setting, we first created a finite element model out of patients’ CT scans (before and after spinopelvic stabilization) and validated its anatomical and biomechanical properties. The patient suffered from a Denis zone I (AO: 61-B2) sacrum fracture. Second, we assessed the effect of the spinopelvic stabilization system on the lumbopelvic area consisting of the vertebra L5, the disc L5/S1, the device itself and the fracture area. The statistical significance of numerical evidence was evaluated by addressing uncertainties in material properties and different levels of osseointegration.

Finally, we validated the impact of an additional CC on this spinopelvic stabilization. We sought to elucidate whether a CC was able to additionally reduce the appearing forces in the sacral fracture area and prevent further dissociation.

## Materials and Methods

### General Features and Subjects

**FIGURE 1:**
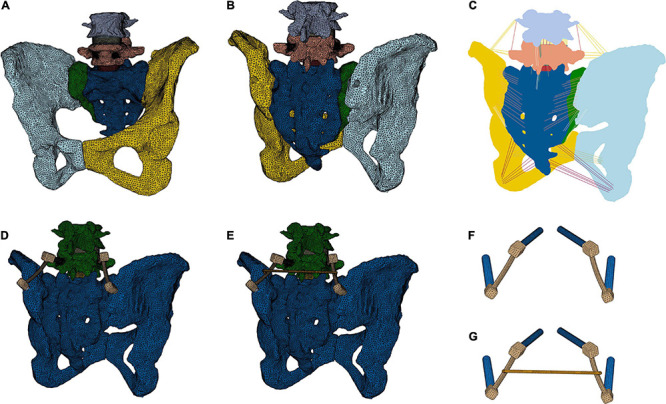
Creation of a finite element model of the lumbopelvic area. The view from anterior **(A)** and posterior **(B,C)** and all ligamentous connections are demonstrated **(C)**. A lumbopelvic stabilization device from L5 to the Os ilium without **(D,F)** and with a cross connector **(E,G)** was modeled.

Finite element models (FE models) were based on computed tomography (CT) images, built from patients from the University Medical Center Goettingen, Germany. CT scans that covered the whole pelvis and L1-L5 were selected for construction of the FE model. The geometries of sacrum and both iliac bones were defined from a patient (preoperatively without device, postoperatively with device including a CC in a nondisplaced sagittal sacrum fracture) who was a 66-year-old man (Figures 1A,B).

To simulate the preoperative spinal movement, all parts of the device have been removed. In analogy, the simulations without CC were conducted on the same geometrical model removing the CC. To easier visualize the models and since the device is implanted in L5, L1-L3 have been cut out and just L4-L5 and the pelvis were considered as bony structures in the numerical model.

The models were generated modeling the intervertebral disks (IVD) with annulus, nucleus and endplates, according to Spina and El Bojairami (El Bojairami et al., 2020; Spina et al., 2020). The ligamentous stabilization was considered taking primary ligament groups into account, which are the interspinous ligament (ISL), anterior sacroiliac ligament (ASL), long posterior sacroiliac ligament (LPSL), short posterior sacroiliac ligament (SPSL), supraspinous ligament (SS), sacrotuberal ligament (ST), iliolumbal ligament (ILL), anterior longitudinal ligament (ALL), interosseous sacroiliac ligament (ISIL), ligamentum flavum (LF), posterior longitudinal ligament (PLL), sacrospinal ligament (SL), and sacrotuberal ligament (TL).

The three models (without device; with device but without CC; with device and CC) were simulated applying three different loading conditions: flexion, right lateral bending, and left axial rotation.

The outcome parameters were range-of-motion (ROM) and maximum stresses. Depending on the area and the quantity of interest, the analyzed stress measures were von Mises stresses and maximum principal stresses (absolute values, Abs), with the latter being the maximum or minimum principal stresses depending on which absolute value is higher. The areas-of-interest were the disk L5/S1, the vertebral body L5, the fixation device and the fracture area. The overall effect of the CC on the disk L5/S1, L5, the fixation device, and the fracture zone was statistically evaluated in different bone qualities.

### Volumes Reconstruction and Surfaces

Computerized tomography images consisted of 0.35 × 0.35 mm resolution and 0.5 mm slide thickness. The images were segmented using a combination of thresholding and manual techniques in MeVisLab 3.0.2 (MeVis Medical Solutions AG, Bremen, Germany) to create the bony geometries. 3D, triangular surface meshes of sacrum, ilium and spinal vertebrae (L4-pelvis) were exported. Since soft tissue can hardly be detected in CT images, the geometries of the intervertebral disks (IVD) were created in Autodesk Inventor 2016, based on the surface geometries and spatial positions of the lumbar bodies as well as considering lumbar anatomy in general. The meshes were then generated in Hypermesh 2019 (Altair Engineering, Troy, MI, United States) using triangular elements.

The facet joints between the vertebrae were considered as face-to-face contact with Coulomb friction and a friction coefficient of 0.1. The intervertebral disks were tied to the vertebra. The geometries and spatial positions of IVD and spatial positions of ligaments were approved by the clinical authors (DS, LW, and WL).

The surfaces of the fixation device and the screws were created in Hypermesh taking the dimensions from the device presented in section “Device.” The screws were tied to the device. Screws have been inserted in the bones by creating holes in the initial geometry (initially reconstructed as intact from CT images) via standard Boolean operations implemented in Hypermesh. The screw thread was not geometrically modeled. For the simulations with the CC, the CC was tied to the device.

### Meshing

The geometries of the bony structures, IVD and fixation device were spatially discretized by means of Lagrange tetrahedral elements with a linear interpolation of the displacement field in a standard Galerkin finite element formulation. All ligament groups (ISL, ASL, LPSL, SPSL, SS, ST, ILL, ALL, ISIL, LF, PLL, SL, and TL) were represented with two-noded truss elements (Figure 1C and Table 1). All meshing operations were performed in Hypermesh.

**TABLE 1:** Material parameter values employed in numerical simulations.

**Part**	**Young’s modulus (MPa)**				**Poisson’s ratio**	**References**	**Element type**
**L4, L5, S1, Pelvis**							
Cortical bone	10,000				0.3	(Panjabi et al., 1992;	C3D6
Trabecular bone	100				0.2	Rohlmann et al., 2006b; Rohlmann et al., 2006a; Zhang et al., 2009; Becker et al., 2020)	C3D4
**Intervertebral disc**							
Endplate	100				0.4	(Zhong et al., 2006; Kurutz and Oroszváry, 2010)	C3D4
Annulus	4.2				0.45	(Zhang et al., 2009)	C3D4
Nucleus	4				0.49	(Lavaste et al., 1992)	C3D4
**Ligaments** (E_*lig*_)	<2.5%	2.5-5%	5%-10%	>10% strain		(Goel et al., 1993)	
ISL	200	285	525	510	0.3		T3D2
ASL	39	55	103	100	0.3		T3D2
LPSL	29	40	75	73	0.3		T3D2
SPSL	13	18	33	34	0.3		T3D2
SS	26	37	68	66	0.3		T3D2
ST	17	24	45	44	0.3		T3D2
ILL	40	57	105	102	0.3		T3D2
ALL	7.8 (<12% strain)			20(>12% strain)	T3D2		T3D2
ISIL	10 (<14% strain)			11.6(>14% strain)	T3D2		T3D2
LF	15 (<6.2% strain)			20 (>6.2% strain)	T3D2		T3D2
PLL	10 (<11% strain)			20 (>11% strain)	T3D2		T3D2
SL	8 (<20% strain)			15 (>20% strain)	T3D2		T3D2
TL	10 (<18% strain)			58.7 (>18% strain)	T3D2		T3D2
**Implant**							
Screws	105,000				0.36		C3D4
Device	210,000				0.29		C3D4
Connector	102,500				0.36		C3D4

To distinguish between cortical and trabecular bone, the mesh of the bony structure was divided into a 1.5 mm thick outer domain for cortical and an interior for trabecular bone structure according to Yamamoto et al. (1989) and Lindsey et al. (2018). To consider different osseointegration levels, a 1.5 mm thick bone layer around the screws with separate material properties was defined.

Overall, each numerical model has been discretized with around 1.062.062 elements, of which 902.944 for the bony structures, 84.965 for the IVD, 73.997 for the fixation devices and 156 for the ligaments. Accordingly, the total number of degrees of freedom of each model is around 1.115.556.

### Material Properties

The model and material properties were set based on previously published literature (Yamamoto et al., 1989; Lindsey et al., 2018). Linear elastic isotropic constitutive models were assigned to both cortical and trabecular bone. The annulus, nucleus, and endplates of IVDs are also modeled as isotropic in agreement with Shin et al. (2007, 2018) and El Bojairami et al. (2020). An isotropic modeling approach is chosen for the fibrosus annulus in the IVD, instead of an anisotropic one, because the directionality of fibers has been shown to have a limited influence on the mechanism of load transfer between vertebrae (Mengoni et al., 2016). The ligaments were modeled as non-linear spring elements using displacement-force load curves derived from the literature (Rohlmann et al., 2006b; Ayturk and Puttlitz, 2011; Finley et al., 2018).

The reference values of parameters employed in numerical simulations (if not differently specified) are listed in Table 1. In order to analyze the robustness of numerical results with uncertainties in parameters’ values (i.e., uncertainties due to patient-specific material properties), we have also performed a campaign of numerical simulations by varying each parameter as reported in Table 2. In the parametric analysis, a single parameter was varied in each simulation and fifteen simulations were performed for each case study.

**TABLE 2:** Material properties uncertainties employed for the parametric analysis.

	**Min (MPa)**	**Middle (MPa)**	**Max (MPa)**	**References**
Cortical bone	5000	10000	12000	Panjabi et al., 1992; Rohlmann et al., 2006a, b; Zhang et al., 2009; Yang et al., 2016; Becker et al., 2020
Endplate	24	100	1000	Benzel, 1999; Jaramillo and Garcia, 2017; Mahato et al., 2019
Nucleus	1	4	10	Lavaste et al., 1992; Chen et al., 2008; Zhang et al., 2009
Annulus	2	4.2	6	Lavaste et al., 1992; Chen et al., 2008; Zhang et al., 2009
Ligaments	0.9 E_lig_	E_lig_	1.1 E_lig_	

### Model Validation

The range of motion obtained in numerical simulations was compared with the one reported in previously published experimental and numerical studies (Yamamoto et al., 1989; Tullberg et al., 1998; Ivanov et al., 2009; Jahng et al., 2013; Dreischarf et al., 2014; Kyaw et al., 2014; Lindsey et al., 2015; Nagamoto et al., 2015; Coombs et al., 2017; Jaramillo and Garcia, 2017; Kibsgård et al., 2017; Joukar et al., 2018; Mahato et al., 2019; Supplementary Figures 1, 4).

### Biomechanical Assessment of Boundary and Loading Conditions

For all models, couples of 10 Nm were applied to produce flexion (in the sagittal plane), right lateral bending (in the frontal plane) and left axial rotation (in the transverse plane) (Yamamoto et al., 1989; Lindsey et al., 2015). The loads were applied using a master node at the middle of the top surface of L4. For realistic mechanical analyses, the force given by the body mass of thorax and head (300 N) – according to Danielson (Danielson et al., 1998) and Sterba (Sterba et al., 2018) – has been incorporated distributing the compressive load on the upper L4 surface in reference normal direction. Zero displacement boundary conditions were introduced in the joint between pelvis and femur. All models were analyzed in Abaqus (Dassault Systèmes, Vélizy-Villacoublay, France) and results were processed in Microsoft Excel (Microsoft Corporation, Redmond, WA, United States).

Three repair strategies have been compared: (1) without fixation, (2) fixation without CC, (3) fixation with CC. Within each of these situations, three loading conditions have been mathematically assessed: (1) anteroposterior bending, (2) lateral bending, and (3) torsion.

### Lumbopelvic Stabilization

The simulated operative procedure was a lumbopelvic stabilization from L5 to the ilium with a pedicle screw on each side of L5 and one screw on each side to the ilium (IS screws), connected with a long rod on each side. These two long rods were connected with CC or not connected to each other (Figures 1D,E). The procedure was simulated according to Kim and Benzel (Benzel, 1999; Kim, 2006). Briefly, a pedicle screw was inserted at the dorsal facet of the mammillary process through the isthmus of the pedicle into the vertebral body without penetrating the spinal canal. A second screw was implanted from the posterior iliac crest, directing ventral and caudal toward the anterior inferior iliac spine. On each side, a long rod was used to connect these two screws (Aebi et al., 2007). All simulated surgeries were approved by the clinical authors (DS, LW and WL) and applied as in the postoperatively performed CT scans.

### Device

The device model is constructed as an approximation of a device delivered by DePuySynthes (Warsaw, Indiana, United States). It consists of two L5 pedicle screws (titanium alloy), two iliac screws (titanium alloy), a rod with cross-link clamps (titanium, stainless steel) and a cross connector (titanium). The dimensions of the model are presented in Supplementary Figure 5. The screws are simplified as cylinders.

### Levels of Osseointegration

Nine diverse levels of osseointegration of the devices have been taken into consideration according to Panjabi et al. (1992), Rohlmann et al. (2006a, b), and Zhang et al. (2009) from low integration (case 1), where the interface between the screws and the bone is equal to cancellous bone (100 MPa), to intermediate integration (cases 2 to 8), where the interface stiffness is in between cancellous and cortical bone (i.e., between 100 MPa and 10.000 MPa), and complete integration (case 9), where the interface is equal to cortical bone (10.000 MPa). For each level of osseointegration, the values of the material constants of the screw-bone interface are listed in Table 3. If not explicitly specified, a level of osseointegration equal to 10.000 MPa (cortical bone) has been employed in the numerical simulation.

**TABLE 3:** Variations of the Young’s modulus (MPa) and Poisson’s ratio of the interface between the screws and the bone for the osseointegration analysis.

**Cases**	**1**	**2**	**3**	**4**	**5**	**6**	**7**	**8**	**9**
Young’s modulus (MPa)	100	250	500	750	1000	2500	5000	7500	10000
Poisson’s ratio	0.2	0.21	0.23	0.24	0.25	0.26	0.28	0.29	0.3

### Range of Motion

**FIGURE 2:**
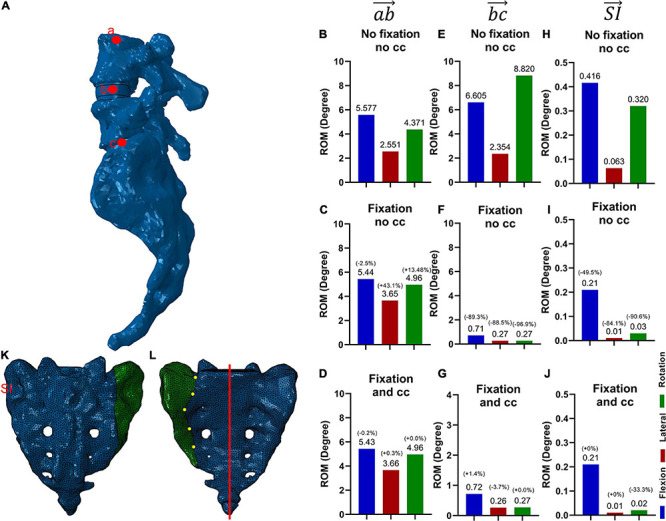
Lumbopelvic axis and three measuring points for range of motion (ROM) **(A)** and corresponding motion (for all three conditions and vector$\underset{\left(ab\right)}{⟶}$**B–D**, vector $\underset{\left(bc\right)}{⟶}$ **E–G**, $\underset{\left(SI\right)}{⟶}$ **H–J**) and addition of a central vertical sacral fracture (Denis zone I, K, L). The range of motion was assessed along the vector $\underset{\left(ab\right)}{⟶}$ for the motion from L4 to L5 without fixation **(B)**, with fixation without **(C)** and with **(D)** the cross connector, whereas the motion from L5 to the sacrum is labeled by the vector $\underset{\left(bc\right)}{⟶}$, again measured without fixation **(E)**, with fixation without **(F)** and with **(G)** the cross connector. The SI ROM is measured by the vector $\underset{\left(SI\right)}{⟶}$, and depicted in **H–J**. A central transalar sacrum fracture, Denis classification zone I (identified by yellow dots), was created to simulate the most common central sacrum fracture type **(K,L)** (Denis et al., 1988). The red line in L depicts the cut surface for Figures 6D–F.

Assessing the resulting range of motion (ROM) after spinopelvic stabilization was thought to deliver information on the impact of the cross connector on spinal mobility. For this purpose, on the middle top of L4 (a), L5 (b) and the sacrum (c), three distinctive measuring (Lagrangian) material points were identified. The angles that were produced by the vector $\underset{\left(ab\right)}{⟶}$ before and after the simulation depicted the range of motion from L4 to L5. The angle produced by the vector $\underset{\left(bc\right)}{⟶}$ before and after the simulation depicted the range of motion from L5 to the sacrum (Figures 2A,B). In analogy to Hammer and Klima, the sacroiliac joint motion was assessed and compared to the values presented in the literature (Supplementary Figure 4; Hammer and Klima, 2019).

### Fracture Model

To examine the effect of the CC on the sacrum fracture itself, a fracture pattern was created in the sacrum, deduced from the most common sacrum fracture (Figures 2K,L). The fracture was simulated by a separation of the elements in the FE mesh along the prescribed pattern. A face-to-face contact with Coulomb friction and a friction coefficient of 0.2 was implemented between the two bony surfaces. In what follows, when referring to the fracture area, the separation surface at the fracture is meant. To assess the effect of the fixation procedure on the fracture area, the interfragmentary movement (IFM) is analyzed. In analogy to Carrera, four points at the fracture side were selected and the displacements due to the applied moments between initially superimposed points were calculated (Carrera et al., 2016; Figure 7).

### Data Analysis

The resulting maximum stresses for each repair strategy (without fixation, fixation without CC, fixation with CC), for all three loading conditions (Flexion, lateral bending and torsion) and for the nine levels of osseointegration (*min* with interface stiffness comparable to cancellous bone, *average* with stiffness between cancellous and cortical bone and *max* with stiffness comparable to cortical bone) were calculated using post-processing routines in Abaqus (Dassault Systèmes, Vélizy-Villacoublay, France).

### Statistical Analyses

Statistical analysis was performed using a two-way analysis of variants (ANOVA)/Mixed Model with post-hoc *t*-test and a Tukey correction for multiple hypothesis testing according to the recommendations of Lakens (Lakens, 2013) with GraphPad Prism version 9.00 (GraphPad Software, Inc.). In the violin plots, the mean, 25th as well as 75th percentile are displayed.

## Results

### Effect of the Fixation Procedure on the Ranges of Motion

The ROM were compared for the L4/L5 movement (Figures 2B–D, vector $\underset{\left(ab\right)}{⟶}$), the L5/S1 movement (Figures 2E–G vector $\underset{\left(bc\right)}{⟶}$), and the sacroiliac joint movement (Figures 2H–J vector $\underset{\left(SI\right)}{⟶}$).

The addition of a fixation device led to a substantial decrease in ROM in the movement from L5 to the sacrum (Figures 2E,F). Similarly, decreased ROM can be seen in the sacroiliac joint (Figures 2H–J). The addition of a CC (Figures 2D,G,J) compared to the situation without the CC (Figures 2C,F,I), did not change the residual ROM substantially. The addition of a fixation did not result in a substantial change in ROM from L4 to L5 (Figures 2B,C,D).

### Model Validation: ROM

We compared the range of motions (ROMs) of our finite element model for each movement with the ones reported on the basis of experimental or numerical studies by Yamamoto et al. (1989); Hungerford et al. (2004), Ivanov et al. (2009); Dreischarf et al. (2014), Kyaw et al. (2014); Lindsey et al. (2015), Nagamoto et al. (2015); Coombs et al. (2017), Hu et al. (2017); Jaramillo and Garcia (2017), Kibsgård et al. (2017); Cross et al. (2018), Joukar et al. (2018), and Mahato et al. (2019) (ROM for L4/L5 and L5/S1: Supplementary Figure 1; ROM for SI: Supplementary Figure 4). In the L4/L5 movement (vector $\underset{\left(ab\right)}{⟶}$), and L5/S1 movement (vector $\underset{\left(bc\right)}{⟶}$), our model was in excellent agreement with the literature for flexion and lateral bending, while showed slightly higher values for axial rotation (Supplementary Figure 1). Considering that the present work addresses a single case study of a diseased patient, the developed finite element model shows biomechanical performance in general agreement with literature data.

### Effect of the Fixation Procedure on the Stresses in the Intervertebral Disc L5/S1

**FIGURE 3:**
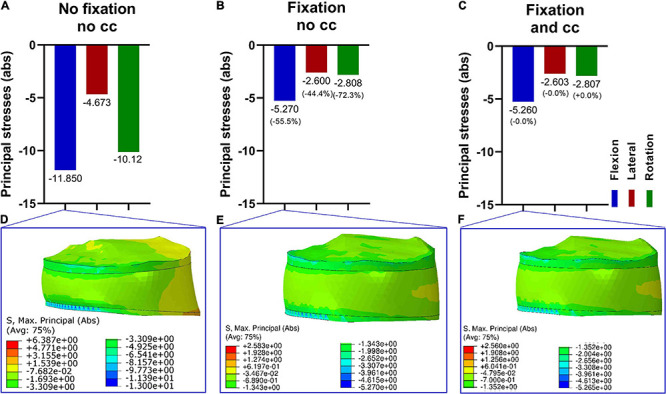
Maximum principal stresses (absolute values) in the intervertebral disk L5/S1 (nucleus pulposus part) in three loading conditions **(A–C)** and corresponding stress distribution exemplarily depicted in the F/E situation **(D–F)**. Without fixation **(A,D)**, the resulting principal stresses were higher compared to the fixation procedure **(B,C,E,F)**. Upon fixation without cross connector **(B,E)** and with cross connector **(C,F)**, the differences appeared to be marginal.

The consequences of the fixational device on the intervertebral disc were firstly evaluated. Since nucleus pulposus and annulus fibrosus showed similar values and tendencies, we decided to just show the nucleus pulposus. Without fixation, the maximum principal stress was the highest in flexion, followed by rotation and lateral bending (Figure 3A,D). After adding a fixation device, the maximum principal stresses were reduced for all loading conditions, and there were no large differences with and without the use of a cross connector (Figures 3B,C,E,F).

### Effect of the Fixation Procedure on the Stresses in the Fifth Vertebra (L5)

**FIGURE 4:**
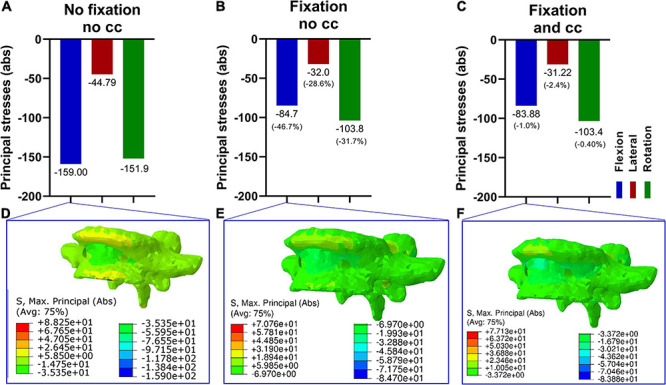
Maximum principal stresses (absolute values) in the fifth vertebra in three loading conditions **(A–C)** and corresponding stress distribution exemplarily depicted in the F/E situation **(D–F)**. Without fixation **(A,D)**, the resulting principal stresses in F/E and rotation were higher compared to the fixation procedure **(B,C,E,F)**. Upon fixation without cross connector **(B,E)** and with cross connector **(C,F)**, the differences appeared to be marginal.

Next, the effects of a lumbopelvic stabilization on the fifth vertebra were assessed in terms of principal stresses (Figure 4). A fixation reduced the maximal principal stresses in flexion and axial rotation by more than 30% and in lateral bending by 28.6%. There were only marginal differences with and without the use of a cross connector (Figures 4B,C,E,F).

### Effect of the Fixation Procedure on the Stresses in the Device

**FIGURE 5:**
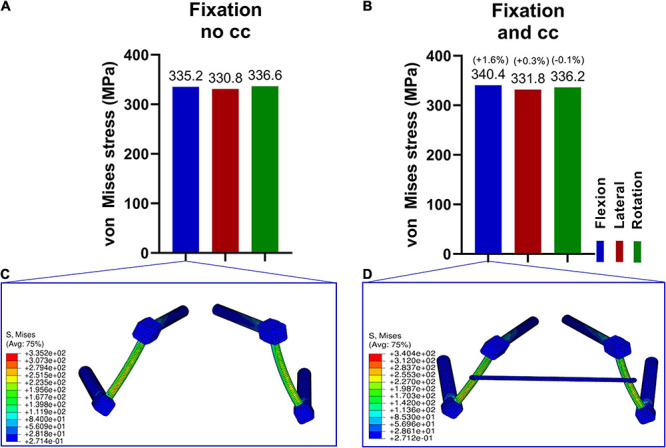
Maximum von Mises stresses in the fixation device in three loading conditions **(A,B)** and corresponding stress distribution exemplarily depicted in the F/E situation **(C,D)**. The stresses in the fixation without cross connector **(A,C)** and with cross connector **(B,D)** are similar for all loading cases.

To guarantee a functional L5/Ilium stabilization and intact cross connector, the maximum “von Mises” stresses on the device were evaluated. In Figure 5, the stresses in the device were assessed with and without the cross connector. The maximum stress was similar in all loading cases and the addition of a cross connector did not lead to substantial lowering of the maximum “von Mises” stresses (Figures 5A–D). Material failure is one reason for post-surgery complications. Thus, the strength of the device is analyzed to ensure the resistance of the device by comparing the stresses in the screws with values reported by Amaritsakul and Shin (Amaritsakul et al., 2014; Shin et al., 2018). In these articles, the strength of pedicle screws (titanium alloy) of multiple shapes and dimensions were compared. Stresses in the herein simulated screws (Supplementary Figure 5B) is in all loading cases much lower than the failure values reported by Amaritsakul (>500 MPa and in the range 1000–3000 MPa for several types of screws) and in the range of the values reported by Shin, that is around 100 MPa (Amaritsakul et al., 2014; Shin et al., 2018). The maximum stresses in the device occurs in the rods. The presented values are in a range in which no failure of such materials is to be expected.

### Effect of the Fixation Procedure on the Fracture Area

**FIGURE 6:**
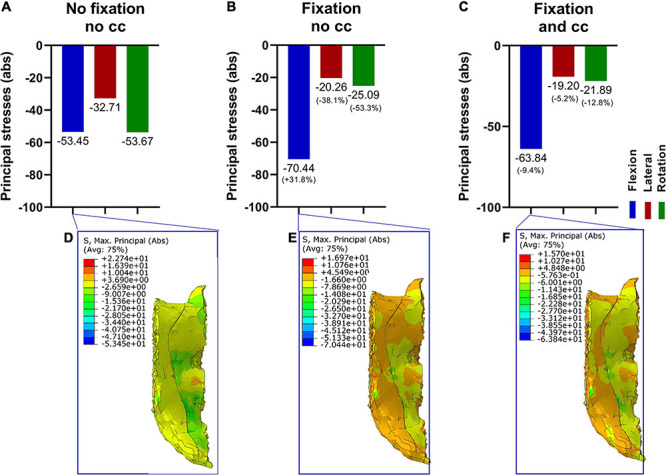
Maximum principal stresses (absolute values) in the fracture area **(A–C)** and corresponding stress distribution exemplarily depicted in the flexion situation **(D–E)**. The fixation alone reduced the maximum stresses in lateral bending and rotation **(A,B,E)**, and a cross connector further reduced the occurring maximum stresses in all loading cases **(C,F)**.

The simulated fracture area was analyzed in terms of the maximum principal stresses and interfragmentary movements (IFM) in all conditions. While the fixation alone reduced the stresses under lateral bending and torsion substantially, the stresses increased in flexion. The addition of a cross connector reduced the stresses in the fracture area for all loading cases (Figures 6A–F).

**FIGURE 7:**
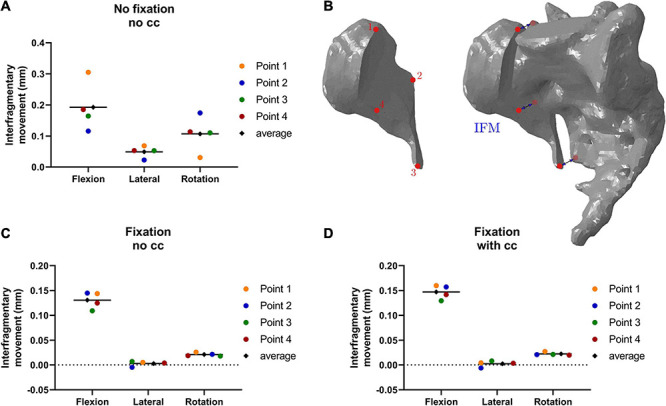
Interfragmentary movement (IFM) in the fracture area in different conditions: Without fixation and cross connector **(A)**, with fixation and no cross connector **(C)** and with fixation and cross connector **(D)**, the points of measurement are depicted in **(B)**. In flexion, the interfragmentary movement is highest in all points and all conditions **(A–D)**. The fixation alone reduced the occurring interfragmentary movement substantially **(C)**, while an additional cross connector led to almost identical values **(D)**.

In addition to the stress analyses, the interfragmentary movement (IFM) was analyzed according to Carrera et al. (2016). The corresponding displacements are presented in Figure 7. The fracture is significantly stabilized by a fixation. In contrast, the effect of the cross-connector is not that evident for the IFM. Overall, the effect of the cross-connector seems to be beneficial in terms of fracture area stress, while the interfragmentary movement is not reduced using a CC.

### Statistical Analysis of Osseointegration Levels

To assess if the obtained differences were attributable to the cross connector, inter-case variability was introduced on the level of osseointegration. This might be relevant since osseointegration affects the way loads are transferred through the device. For this purpose, nine different levels of osseointegration (from low = 100 MPa/low bone quality/spongious bone to high = 10.000 MPa/high bone quality/spongious bone, Table 3), have been assessed according to Becker et al. (2020).

A two-way analysis of variance (ANOVA) was conducted to estimate whether differences between the two conditions (with and without cross connector [CC]) were effective or apparent. If so, a post-hoc *t*-test was performed to quantify these differences. The analysis addresses the axial rotation case.

**FIGURE 8:**
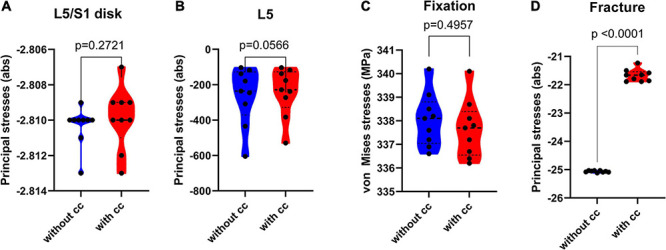
Violin plots depicting the maximum principal stresses (absolute values) in the disk L5/S1 **(A)**, fifth lumbar vertebrae **(B)** and fracture area **(D)** and the von Mises stresses in the fixation device **(C)** in MPa, statistically validated in different variations of osseointegration (100 MPa to 10.000 MPa) for the axial rotation case. In the disk area L5/S1, the resulting effect of a cross connector was not significant [*F*(1,8) = 1.391, *p* = 0.2721, SS = 8.89*10^-7, δ = 0.0004, subplot A]. In the lumbar vertebra L5 [*F*(1,8) = 4.955, *p* = 0.0566, SS = 1842, δ = 20.23, subplot B] and fixational device [*F*(1,8) = 100, *p* < 0.0001, SS = 0.68, post-hoc t-test *t*(16) = 0.6971, *p* = 0.4957, δ = 0.39, subplot C], the effect of a cross connector was not significant. In the fracture area, the resulting principal stresses (subplot D) were significantly lower after the addition of a cross connector [*F*(1,8) = 2066, *p* < 0.0001, SS = 51.78, post-hoc t-test *t*(16) = 46.28, *p* < 0.0001, δ = 3.392] (Mean, 25th and 75th percentile).

The addition of a cross connector did not increase the resulting stress in the disk L5/S1 (Figure 8A *p* = 0.2721), and differences in L5 and the fixational device were not significant as well (Figure 8B *p* = 0.0566 and Figure 8C, *p* = 0.4957, respectively). On the other hand, in the fracture area, the cross connector reduced the occurring principal stresses significantly, with a difference on mean values corresponding to around a 10% variation (Figure 8D, *p* < 0.0001, δ = 3.392).

### Statistical Analysis of Material Properties Uncertainties

In order to investigate if the afore-traced comparison between different fixation procedures were robust also with respect to uncertainties in material properties, additional finite element analyses were conducted for the axial rotation case by varying material properties within the ranges reported in Table 2. Therefore, an ANOVA test was performed on the biomechanical outcomes obtained with and without the cross connector.

Firstly, the effect on the stabilization obtained by the fixation procedure was assessed in terms of range of motion. Results (Supplementary Figure 2) indicate that, although the addressed variation of material properties might change the ROM up to 30%, the comparison of ROM between the case without CC and with CC is unaffected.

Moreover, stresses in the IVD L5/S1, L5, fracture and fixation are investigated. Results (Supplementary Figure 3) indicate that, despite the wide variations of model parameters, the stress comparison between the case without and with CC is not affected by such variations in the L5/S1 disk (Supplementary Figure 3A, *p* = 0.2806). However, the differences with and without the CC were small, but significant in L5 and the fixation device (L5: Supplementary Figure 3B, *p* < 0.0001, δ = 0.446, fixation: Supplementary Figure 3C, *p* = 0.0024, δ = 0.473). Comparable to the results from different osseointegration levels, contrasting different material properties likewise showed a significant difference in the fracture area with and without the CC, with about 10% difference on mean values (Supplementary Figure 3D, *p* < 0.0001, δ = 2.727).

## Discussion

The unstable sacrum fracture needs operative reduction and biomechanical stability in order to heal properly. The naturally high complication and infection rate after operative procedures in this anatomical location demands smallest possible incisions yet providing most stability (Bellabarba et al., 2006).

Surgery of the lower spine and pelvis faces the difficulty of disparate patient cohorts: The young patient with proper bone parameters and the osteoporotic patient suffering a fragility fracture. Absolute stability of the surgical treatment is paramount in both. The more complex a sacrum fracture (or spinopelvic dissociation) gets, the more elaborate the fixation needs to be. Beginning with simple percutaneous iliosacral screws for uncomplicated sacrum fractures, the H-shaped sacrum fracture requires sophisticated spinopelvic stabilization with pedicle screws in L4 or L4 and L5 and a sacral-alar-iliac fixation, both of which are connected with a vertical rod. With such a bilateral construct, the applying vertical forces are adequately addressed (Jazini et al., 2017a). The armamentarium of spinal surgery also contains the possibility of adding a cross connector to these bilateral rods.

We created a finite element model to verify the stabilizing effect of a spinopelvic device (two 7.0 mm L5 screws, two 9.00 mm iliac screws) and assess the accessory effect of a CC in this anatomical area. Therefore, three load cases, compression with flexion, with right lateral bending and with left axial rotation, were investigated for three lumbar models (without fixation, with fixation without CC, with fixation with CC).

The ROM in the IVD L5-S1 and the SI joint are significantly reduced (49.5–96.9%) when a fixation is used. In contrast, the reduction of the ROM in the IVD L4-L5 is not that evident. The ROM in lateral bending even shows a contrary trend and increases due to the use of a fixation. The differences in the ROM of the IVD L4-L5 to the ROM in IVD L5-S1 and the SI joint are due to the position of the device. Since the pedicle screws are fixated in the L5, only the lower spinal part is stabilized. In all three locations, the additional use of a CC could not or only marginally further reduce the ROM. Only the ROM in the SI joint in axial rotation was further reduced by 33.3 % compared to the ROM using a fixation without CC which corresponds to another 3 % reduction compared to the original ROM without fixation.

Plenty of biomechanical studies for the analysis of spinal movement are available, out of which a few in particular analyzed the effect of a cross connector. In a biomechanical evaluation of zone 2 sacral fractures (as opposed to the zone 1 fracture considered in this study), the effect of a lumbopelvic fixation was analyzed in fifteen frozen cadaveric specimens by Jazini et al. (2017a). A transverse cross-connector, in combination with an anterior plate increased the pelvic ring stability especially in axial rotation and not in lateral flexion, but just if there was a “small-gap fracture model.” Likewise, the addition of a cross connector did not add to the principal stresses in lateral bending in our study in the L5/S1 disk. Even for zone 1 fractures like we demonstrated, the fracture zone had to suffer from significantly less maximum stress, in particular in axial rotation, when a cross connector was implemented. The developed finite element model shows a good comparability to the biomechanical assessment from Jazini et al. (2017a) expanding their findings to zone 1 fractures.

Similarly, (Denis) type 2 fractures were assessed by Acklin et al. in 16 pelves. The authors compared different fixation techniques and a “double plating” method reduced the axial stiffness significantly compared to sole SI screws or a monolateral triangular stabilization (Acklin et al., 2018). Similar to a “double plating,” the present study showed that the cross connector enhanced axial stiffness especially in the fracture area, while they do not refer to lateral bending or flexion.

Korovessis et al. (2001) applied one or two pedicle screw-rod constructs onto a polymethylmethacrylate block system in order to analyze differences in ROM due to the addition of rod-rod cross-links. The authors found a small, but measurable reduction in flexion and extension, but none in lateral bending. The whole construct was assessed, while we saw only minor differences in lateral bending after the addition of a cross connector. We saw the same nonsignificant differences in lateral bending within our construct.

Serhan and Slivka used a corporectomy model with polyethylene blocks to simulate the biomechanical properties of the lumbar spine. They found that regarding torsional stiffness, the implementation of one transverse connector enhanced stability by 45% (Melkerson, 2003). In the present FE model, the reduction of axial rotation was small when a cross connector was added after the usual stabilizing device in L5/S1, but substantial in the fracture area. Since the fracture itself needs stabilization for healing purposes, this area is of particular interest.

Decker et al. introduced a new minimally invasive stabilization technique for lumbosacral fractures, which yielded persuasive results in 10 patients using a L3/L4 and iliac screw with long rods, connected with a cross connector delivering “high construct rigidity,” which we sought to verify with finite element analysis (Decker et al., 2019).

Lumbar fixation methods have often been assessed biomechanically. In a study on ten calf lumbar spines, one-level (L3-L4) and two-level (L2–L4) fixations with and without a transfixator were compared. For one-level constructs, the ROM was reduced in flexion and axial rotation, but not extension and lateral bending. We could not see additional effects of the transverse connector in L5/Ilium in flexion and lateral bending as well. In their study, and for two-level constructs, the ROM in flexion, extension and lateral bending was just slightly reduced, whereas axial rotation was dramatically reduced (Lim et al., 1996). Again, in our FE model, the effects on axial rotation were marginal, while the construct itself was more stable (in the sense of lower principal stresses) after a cross connector was added. In addition, the fracture area itself was stabilized substantially by a cross connector in both lateral flexion and axial rotation in our study.

Since cross-links can be designed differently, the diameter has been demonstrated to directly influence the stability of the construct: Comparing different cross-link brands regarding torsional motion and stiffness in L3-L4 stabilization, Dick et al. (1997) found no statistically significant differences for one or two cross-links in all movements (axial, flexion-extension, and lateral-flexion), but torsional loading, where every cross-link provided significantly more stiffness with an increase of 44% for one cross-link and a proportional effect of the cross-sectional area of the cross-link to the magnitude of increase in torsional stiffness (Dick et al., 1997). We used a transverse connector of 3.5 mm external diameter (9.62 mm^2^), which lower compared to the four devices tested by Dick et al. Compared to their largest cross-link (50.27 mm^2^ cross-sectional area), the one we used was clearly smaller and might be the reason for the moderate effect of our measurements with the cross connector on the axial stiffness, especially in L5/S1 and axial rotation. Within flexion-extension stiffness, Dick et al. could not find significant differences after the addition of a cross connector within their construct, which is in agreement with our findings.

Similar results have been published by Carson et al., combining experimental (instrumented spine segments) with finite element methods. The beneficial effect of a transfixated longitudinal spinal construct has been demonstrated for a one-level fixation. Axial and lateral loading were stabilized by a transfixation of bi-level constructs reducing the stress on internal components of force and moment (Carson et al., 1990). The construct itself has been stabilized by a cross connector in our study as well. In accordance to the study by Carson, this effect was most prominent in axial rotation.

Partially contrary to that, and especially in a long stabilizational device, in a biomechanical fresh-frozen cadaveric study, thoracal stabilizational devices after pedicle subtraction osteotomy (PSO) from T4-T10 were assessed, and one transverse connector had no additional stabilizing effect on flexion/extension, lateral bending or axial rotation (ROM) (Lehman et al., 2015). A PSO leads to substantial loss of integrity in a spinal segment, which is why it cannot be directly compared to a fracture model. Interestingly, these authors could see no stabilizing effect of an additional transverse connector, which we could detect in the fracture area for lateral bending and rotational stress.

The finite element model developed in this study indicates more stability in the fracture area with a transverse connector, especially in rotational movements and when variations of osseointegration are implemented. This could provide the basis for a faster healing of the bony area, but needs prospective clinical validation.

In addition, for more conclusive considerations, the finite element model shall be improved, for instance, by considering a longer spine segment, by improving material modeling approaches (i.e., considering an anisotropic behavior for the annulus fibrosus in the IVD and/or for bony structures), or by considering different fracture patterns. Finally, a validation on several case studies, possibly including a patient-specific assessment of material properties, should be conducted in the future.

## Conclusion

By means of a computational study based on the finite element method, the results of the present work suggest that the cross connector did not ameliorate the range of motion in L4/L5 or L5/sacrum.

A fixation (with or without cross connector) reduced the occurring stresses in the disk L5/S1, vertebral body L5 and the fracture area. Moreover, considering the combination of all loading cases, the presence of a cross connector reduced the maximum stress in the fracture area of around 10%. This difference has been statistically evaluated by considering uncertainties in material properties and different levels of osseointegration (different qualities of the interface bone) in the axial rotation case (significant reduction of maximum stress in the fracture area, *p* < 0.0001).

## Data Availability Statement

The raw data supporting the conclusions of this article will be made available by the authors, without undue reservation.

## Author Contributions

DS, WL, MM, and MG designed the study. MM, MG, and FL performed all experimental procedures. DS, MM, MG, and FL carried out the data analysis. DS, MG, and MM wrote the manuscript. WL, LW, and PW critically revised it for important intellectual content. All authors have approved the final version of the article.

## Conflict of Interest

The authors declare that the research was conducted in the absence of any commercial or financial relationships that could be construed as a potential conflict of interest.

## Supplementary Material

Supplementary Figure 1 Validation of the FE model. The L4/L5 segment showed comparable flexion and lateral bending ranges of motion compared to the literature **(A,B)**, while the axial rotation appeared higher **(C)**. The same occurs for the L5/S1 segment **(D–F)**.

Supplementary Figure 2 Effect of variation of parameters on ROM in axial rotation. The L4/L5 **(A)** segment showed comparable ROM when the material parameters were varied (L4/L5 ROM: with^∗^without CC *F*(1,14) = 1.000, *p* = 0.3343, SS = 1.333^∗^10^-5, δ = 0.001). The L5/S1 segment **(B)** similarly depicted a comparable ROM in the groups without and with CC (L5: with^∗^without CC *F*(1,14) = 3.027, *p* = 0.1038, SS = 5.333 ^∗^10^-5δ = 0.003) (Mean, 25th and 75th percentile).

Supplementary Figure 3 Effect of parameters on principal stresses (absolute values) for L5/S1 disk, L5 and fracture area and von Mises stresses for the fixation device in MPa (axial rotation). **(A)** In L5/S1, the variation of parameters did not significantly affect the maximum stresses in without and with cc groups (L5 disk stress: with^∗^without CC *F*(1,14) = 1.260, *p* = 0.2806, SS = 5.333^∗^10^-5, δ = 0.0027. In L5 **(B)**, the changes of parameters led to a marginal, but statistically relevant difference between the “without cc” and “with cc” group (L5: with^∗^without CC *F*(1,14) = 73.94, *p* < 0.0001, SS = 1.408, post-hoc *t*-test *t*(14) = 8.599, *p* < 0.0001, δ = 0.4333). In the fixation device **(C)**, the differences similarly appeared to be marginal, but significant (Fixation stress: with^∗^without CC *F*(1,14) = 13,72, *p* = 0.0024, SS = 1,680, post-hoc *t*-test *t*(14)3.704, *p* = 0.0024, δ = 0.473). In the fracture area **(D)**, the differences between the group with and without the CC appeared to be significant (Fracture stress: with^∗^without CC Fracture stress: with^∗^without CC *F*(1,14) = 194.5, *p* < 0.0001, SS = 61.06, post-hoc *t*-test *t*(14) = 13.95, *p* < 0.0001, δ = 2.853) (Mean, 25th and 75th percentile).

Supplementary Figure 4 Validation of the FE model in the sacroiliac joint (SI). The SI segment showed comparable flexion **(A)**, lateral bending **(B)**, and axial rotation **(C)** ranges of motion compared to the literature.

Supplementary Figure 5 Structural parameters of the spinopelvic device. The L5 pedicle screw has a diameter of 7 mm, the iliac screw of 9 mm, with a length of 45 and 90mm, respectively. The cross connector has a length of 96.50 mm and diameter of 3.50 mm. The cylindrical cross connector is connected to the two screws with cross link clamps **(A)**. In addition, the maximum von Mises stresses in lateral bending with CC within the screws is shown **(B)**.
