# Supplementary figures and images for: Biomechanical Effects of a Cross Connector in Sacral Fractures – A Finite Element Analysis
Source: Front Bioeng Biotechnol. 2021 May 26;9:669321. doi: 10.3389/fbioe.2021.669321 (PMC8188498; doi:10.3389/fbioe.2021.669321)

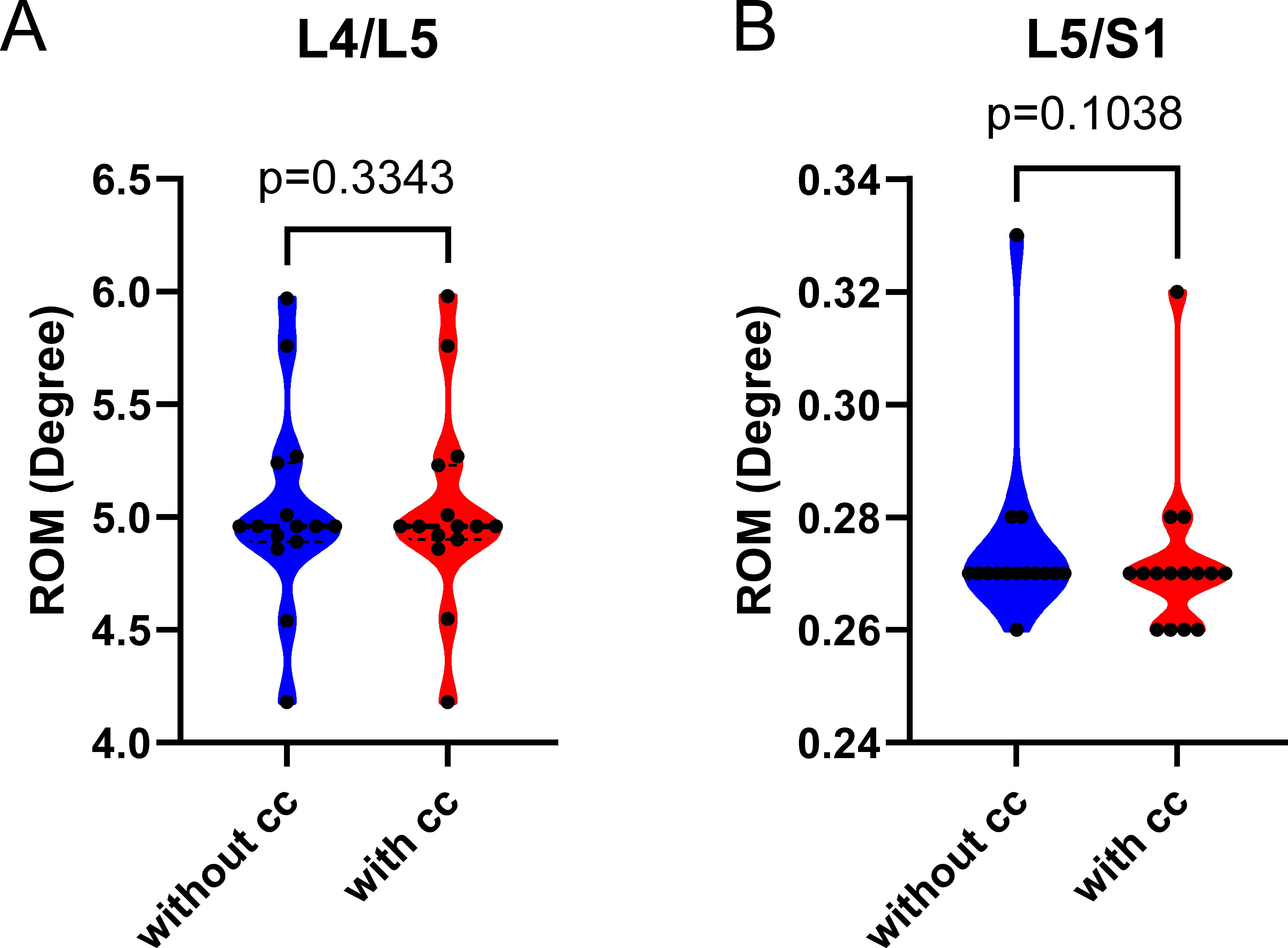

Supplement: Supplementary Figure 2 — Effect of variation of parameters on ROM in axial rotation. The L4/L5 (A) segment showed comparable ROM when the material parameters were varied (L4/L5 ROM: with∗without CC F(1,14) = 1.000, p = 0.3343, SS = 1.333∗10^-5, δ = 0.001). The L5/S1 segment (B) similarly depicted a comparable ROM in the groups without and with CC (L5: with∗without CC F(1,14) = 3.027, p = 0.1038, SS = 5.333 ∗10^-5δ = 0.003) (Mean, 25th and 75th percentile). [file Image_2.JPEG]

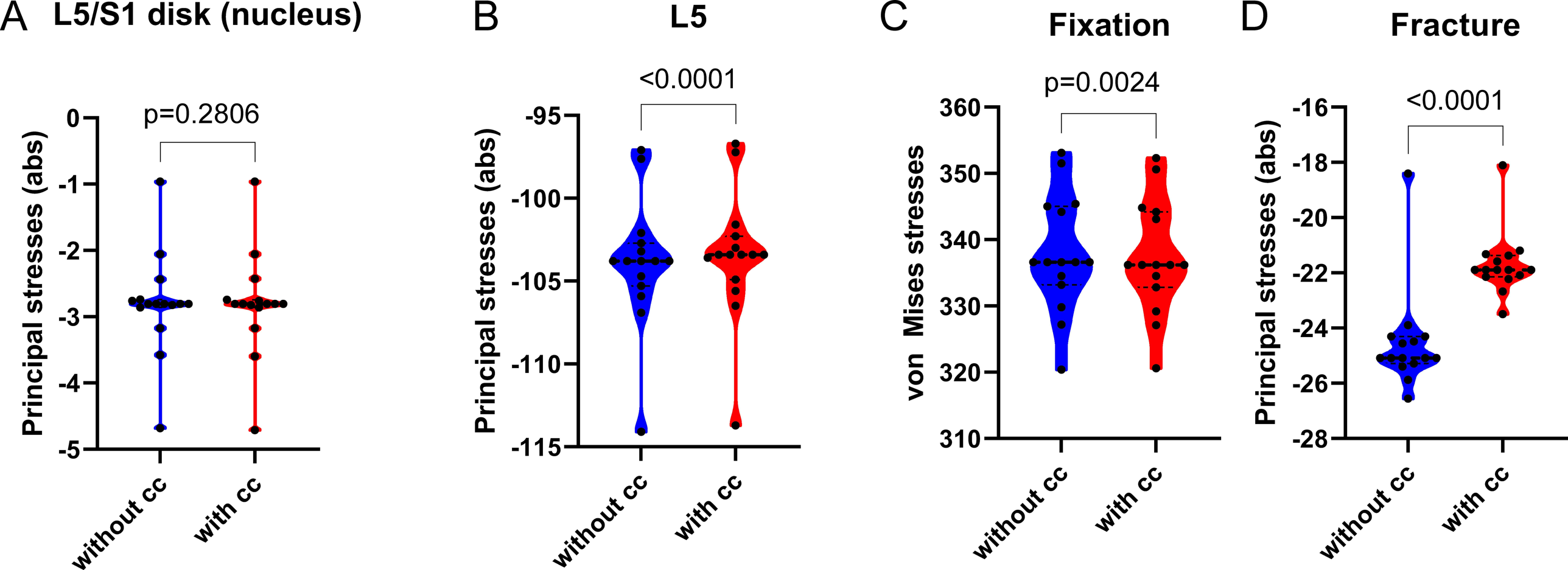

Supplement: Supplementary Figure 3 — Effect of parameters on principal stresses (absolute values) for L5/S1 disk, L5 and fracture area and von Mises stresses for the fixation device in MPa (axial rotation). (A) In L5/S1, the variation of parameters did not significantly affect the maximum stresses in without and with cc groups (L5 disk stress: with∗without CC F(1,14) = 1.260, p = 0.2806, SS = 5.333∗10^-5, δ = 0.0027. In L5 (B), the changes of parameters led to a marginal, but statistically relevant difference between the “without cc” and “with cc” group (L5: with∗without CC F(1,14) = 73.94, p < 0.0001, SS = 1.408, post-hoc t-test t(14) = 8.599, p < 0.0001, δ = 0.4333). In the fixation device (C), the differences similarly appeared to be marginal, but significant (Fixation stress: with∗without CC F(1,14) = 13,72, p = 0.0024, SS = 1,680, post-hoc t-test t(14)3.704, p = 0.0024, δ = 0.473). In the fracture area (D), the differences between the group with and without the CC appeared to be significant (Fracture stress: with∗without CC Fracture stress: with∗without CC F(1,14) = 194.5, p < 0.0001, SS = 61.06, post-hoc t-test t(14) = 13.95, p < 0.0001, δ = 2.853) (Mean, 25th and 75th percentile). [file Image_3.JPEG]

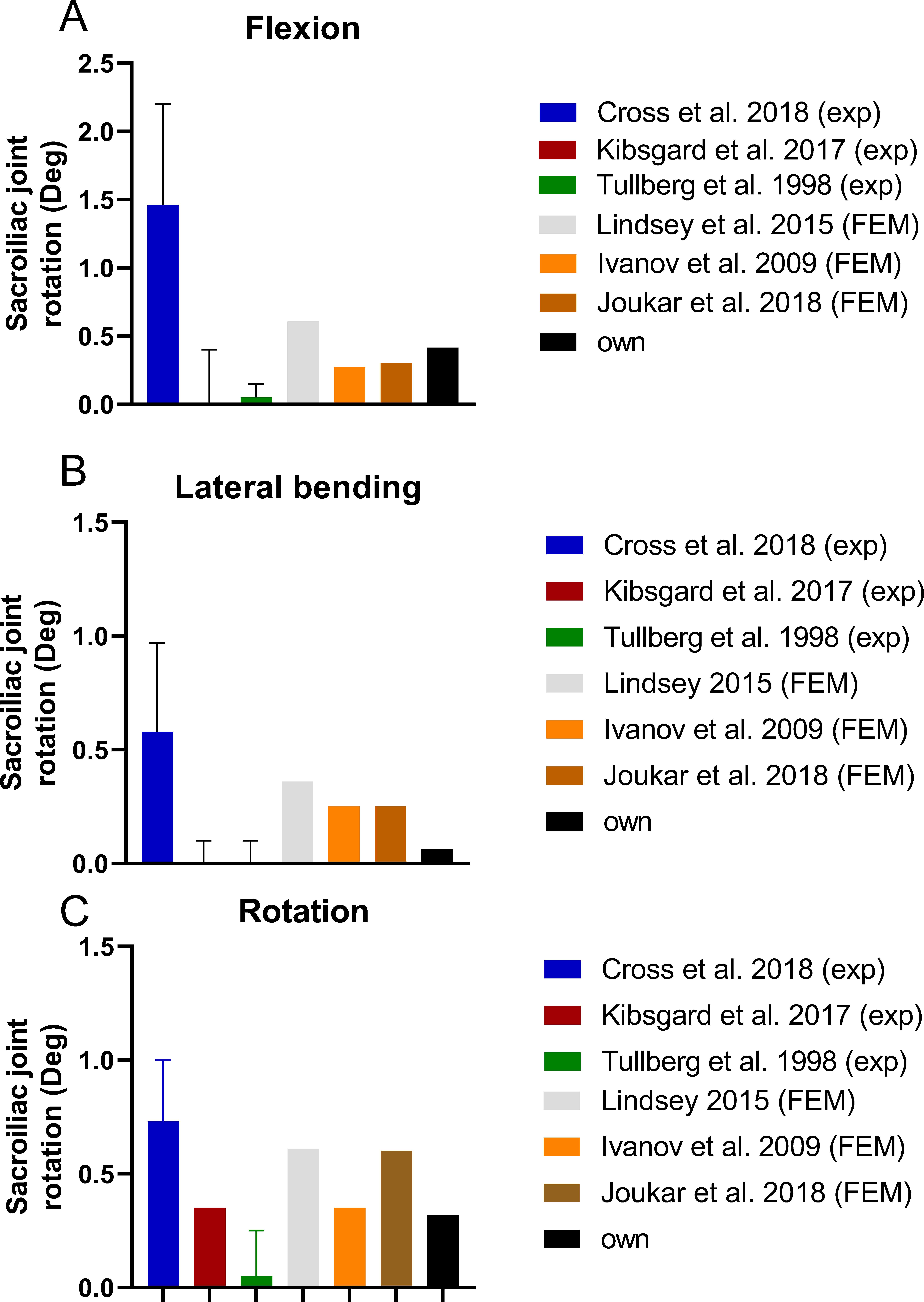

Supplement: Supplementary Figure 4 — Validation of the FE model in the sacroiliac joint (SI). The SI segment showed comparable flexion (A), lateral bending (B), and axial rotation (C) ranges of motion compared to the literature. [file Image_4.JPEG]
